# Discovering biological connections between experimental conditions based on common patterns of differential gene expression

**DOI:** 10.1186/1471-2105-12-381

**Published:** 2011-09-27

**Authors:** Adam C Gower, Avrum Spira, Marc E Lenburg

**Affiliations:** 1 Bioinformatics Program, Boston University, 44 Cummington Street, Boston, Massachusetts, 02215, USA; 2 Division of Computational Biomedicine, Department of Medicine, Boston University School of Medicine, 72 East Concord Street, Evans 631, Boston, Massachusetts, 02118, USA

## Abstract

**Background:**

Identifying similarities between patterns of differential gene expression provides an opportunity to identify similarities between the experimental and biological conditions that give rise to these gene expression alterations. The growing volume of gene expression data in open data repositories such as the NCBI Gene Expression Omnibus (GEO) presents an opportunity to identify these gene expression similarities on a large scale across a diverse collection of datasets. We have developed a fast, pattern-based computational approach, named openSESAME (Search of Expression Signatures Across Many Experiments), that identifies datasets enriched in samples that display coordinate differential expression of a query signature. Importantly, openSESAME performs this search without prior knowledge of the phenotypic or experimental groups in the datasets being searched. This allows openSESAME to identify perturbations of gene expression that are due to phenotypic attributes that may not have been described in the sample annotation included in the repository.

To demonstrate the utility of openSESAME, we used gene expression signatures of two biological perturbations to query a set of 75,164 human expression profiles that were generated using Affymetrix microarrays and deposited in GEO. The first query, using a signature of estradiol treatment, identified experiments in which estrogen signaling was perturbed and also identified differences in estrogen signaling between estrogen receptor-positive and -negative breast cancers. The second query, which used a signature of silencing of the transcription factor p63 (a key regulator of epidermal differentiation), identified datasets related to stratified squamous epithelia or epidermal diseases such as melanoma.

**Conclusions:**

openSESAME is a tool for leveraging the growing body of publicly available microarray data to discover relationships between different biological states based on common patterns of differential gene expression. These relationships may serve to generate hypotheses about the causes and consequences of specific patterns of observed differential gene expression. To encourage others to explore the utility of this approach, we have made a website for performing openSESAME queries freely available at http://opensesame.bu.edu.

## Background

Genome-wide gene expression microarrays have found widespread use because of their high throughput and ability to measure the expression of tens of thousands of genes simultaneously. This technology has made it possible to perform genome-wide searches for changes in gene expression in response to perturbations such as gene knockouts [1] and treatment with bioactive compounds [2]. It has also been useful in identifying gene expression differences associated with histologic subtypes of disease [3], clinical diagnosis [4], prognosis [5], or the efficacy of various therapeutic strategies [6]. However, a challenge for scientists performing genome-wide gene expression microarray analysis has been using these data to generate hypotheses about biological processes responsible for the patterns of differential gene expression associated with a particular trait or experimental variable.

One approach to this problem is to create sets of genes with common biological characteristics, such as chromosomal location, biochemical function, or observed differential expression in some experimental condition, and then to determine whether the genes in these sets are coordinately induced or repressed. If a preponderance of genes in any of these predefined groups of genes (gene sets) is coordinately differentially expressed, it may be reasonable to hypothesize that the biological characteristic upon which that gene set was defined is relevant to the experimental perturbation. Within this broad strategy, two computational approaches are commonly employed. The first uses tests of proportion to determine whether a significant fraction of the genes in a gene set are among those that have been identified as differentially expressed (for examples, see [7,8]). A second approach uses tests of distribution to determine whether the members of a gene set are overrepresented at either extreme of the list of all genes ranked by their degree of differential expression (exemplified by [9]).

The utility of these approaches for gaining insight into the underlying causes of changes in gene expression depends on the availability of defined gene sets that reflect the transcriptional consequences of relevant biological processes. It would therefore be useful to leverage the ever-growing body of transcriptional profiles that have been deposited in freely accessible data repositories, as they represent an enticing source of information about transcriptional responses to a vast number of experimental and biological perturbations. For example, large compendia of gene expression data have been used to predict the function of uncharacterized genes based on their co-expression with genes of known function across diverse experimental conditions (for examples, see [1,10]). Such compendia can also be leveraged to identify conditions that give rise to a given pattern of gene expression.

One way to do this is to collect sets of differentially expressed genes identified in each of the experiments contained in a compendium and to use them in tests of proportion or distribution in a new experiment as described above (for an example, see [11]). Another strategy is to create a catalog of phenotypic or experimental comparisons in a repository of microarray data, and to use tests of proportion or distribution to determine whether any genes observed to be coordinately differentially expressed in a given experiment are also coordinately differentially expressed in any of these comparisons (for examples, see [2,12-15]). An especially interesting example of this approach is the Connectivity Map (CMap) [2]. The CMap consists of a dataset of ranked changes in gene expression associated with the treatment of various cell lines with a large number of compounds (relative to vehicle-treated control samples), and an algorithm for examining the distribution of query genes within each ranked list. This approach was used by its original authors to identify gedunin as an inhibitor of Hsp90, and has since been used to identify the mode of action of natural compounds [16] and to identify compounds that are candidates for preventing [17] or treating [18] lung cancer.

A fundamental challenge of these approaches is that they depend on explicit phenotypic comparisons to define gene sets and/or rank genes according to their differential expression. This requires knowledge of all potentially meaningful comparisons in each dataset and calculating the degree of differential expression associated with each of them. A different approach would be to use a gene expression signature defined by a phenotypic comparison of interest to identify experiments in which those genes exhibit similar coordinate differential expression. An advantage of this approach is that it would not be dependent on prior knowledge of phenotype and would not require an exhaustive catalog of possible phenotypic comparisons. We have developed a method named openSESAME (Search of Expression Signatures Across Many Experiments) to explore the utility of this type of approach.

## Results

### openSESAME Algorithm

**Figure 1:**
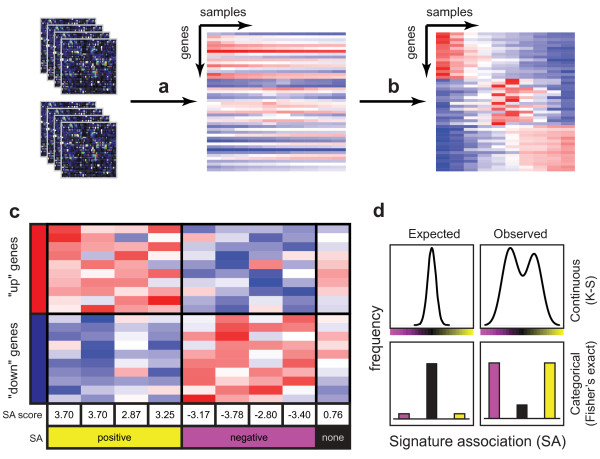
**Identifying association with a gene expression signature using openSESAME**. a) Raw CEL files are normalized to obtain gene-level expression measurements. Here, red and blue indicate genes with high or low levels of absolute expression, respectively. b) Within each experiment, expression measurements for each gene are *z*-score normalized to produce relative expression levels. Here, red and blue indicate high or low expression relative to the other samples in the experiment. c) For each sample in each experiment, a Wilcoxon test is performed between the relative expression values of the "up" and "down" gene sets defined by the query signature, yielding a signature association (SA) score that varies from positive (yellow) to negative (magenta). d) A Kolmogorov-Smirnov (top) or Fisher's exact (bottom) test is performed to determine whether the distribution of SA scores in each experiment is significantly different from the distribution of SA scores across all samples in the repository.

openSESAME (see Figure 1 and Methods) is a method for rapidly identifying datasets in which the expression of a given set of genes is coordinately perturbed. The openSESAME algorithm requires two inputs: 1) a query signature consisting of "up" and "down" gene sets, representing genes that are coordinately induced or repressed, respectively, in some experimental condition, and 2) a collection of normalized gene expression profiles (samples), organized into groups (datasets), within which gene expression levels have been normalized relative to all samples in each group. For the current work, we have used a collection of 75,164 human samples profiled on the Affymetrix HG-U133 family of microarrays and obtained from the Gene Expression Omnibus (GEO) public microarray repository for our database of gene expression data. We have chosen to use this collection of data because it consists of gene expression measurements from a large and diverse body of phenotypes and experimental conditions measured with a common set of oligonucleotide probes using similar technology. Although comparison of differential gene expression across more distinct array platforms is possible [19], we chose to restrict our analysis to data from a single family of related arrays to avoid challenges in data comparability.

A *signature association (SA) score *is calculated for each sample using a Wilcoxon rank-sum test to compare the normalized expression levels of the "up" and "down" genes. The SA score reflects the degree and direction of coordinate perturbation of the expression of the genes in the query signature. If the distribution of relative expression levels of the "up" genes is significantly higher or lower than that of the "down" genes, that sample is given a positive or negative SA score, respectively, and is said to be positively or negatively associated with the query signature. Each dataset is then assessed to determine whether the distribution of SA scores for those samples differs significantly from that expected by chance. Datasets that are enriched in samples that are strongly associated with the query signature are hypothesized to contain an experimental or physiological condition that is biologically related to that used to define the original query signature.

### openSESAME identifies experiments related to estrogen signaling in GEO using a signature of E2 treatment

**Figure 2:**
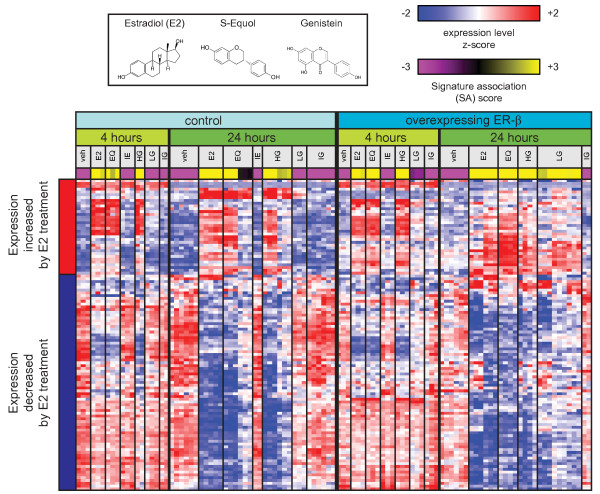
**A gene expression signature of E2 treatment is associated with phytoestrogen treatment in MCF7 cells**. GEO series GSE9936 was identified by openSESAME as significantly enriched in samples associated with the E2 treatment signature. veh, vehicle; E2, 6 nM 17-β-estradiol; EQ, 300 nM S-Equol; IE, 300 nM S-Equol + 3 μM fulvestrant; HG, 300 nM genistein; LG, 6 nM genistein; IG, 300 nM genistein + 3 μM fulvestrant.

We first identified datasets (termed "series" in the GEO nomenclature) from experiments with perturbations or biological phenotypes relevant to estrogen signaling by querying the normalized GEO data with a gene expression signature of treatment of MCF7 cells with the estrogen 17-β-estradiol (E2) [20] (Additional File 1). The series with the most significant enrichment were those in which MCF7 cells were treated with compounds with estrogenic activity (Additional File 2). In these series, samples treated with E2 or other estrogens were positively associated with the E2 treatment signature while samples treated with vehicle, non-estrogenic compounds or antiestrogens were negatively associated with this signature. For example, in series GSE9936 [21], the E2 treatment signature is positively associated with MCF7 cells that have been treated with E2 or with the phytoestrogens genistein or S-Equol, and negatively associated with treatment with vehicle or with the estrogen receptor (ER) antagonist fulvestrant (Figure 2). Furthermore, the SA scores of genistein-treated samples varied with treatment dose and time, and were markedly higher in cells overexpressing ER-β, the preferred receptor for genistein.

**Figure 3:**
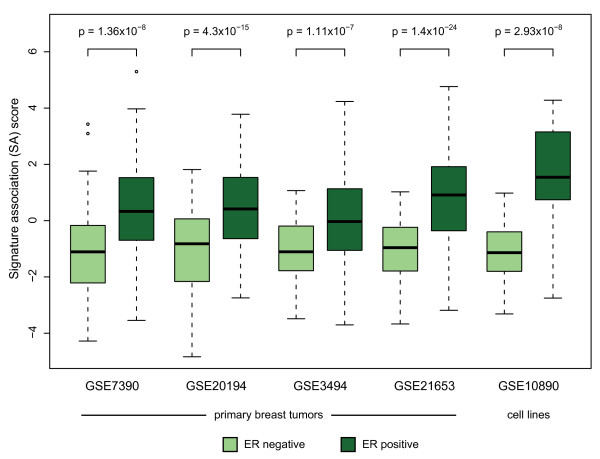
**A gene expression signature of E2 treatment is associated with ER-positive breast cancer**. The degree of association with the E2 treatment signature (SA score) is significantly higher in ER-positive (dark green) than in ER-negative (light green) primary breast tumors [34,53-55] or cell lines. ER status of tumors was extracted from sample annotation provided by each dataset; ER status of cell lines was obtained from the American Type Culture Collection (ATCC) or prior reports [56-61]. Cell lines EVSA-T and UACC-812 were removed from analysis due to conflicting reports regarding ER status. Cell lines MDA-MB-435 and MT-3 were removed from analysis as they have been shown to be cross-contaminated with melanoma and colon cancer cell lines, respectively [62,63]. Heavy lines and shaded boxes indicate the median and interquartile range (IQR) of the SA scores of each group, respectively. Dashed lines extend to the most extreme data points within 1.5 * IQR of the shaded box. Points outside the dashed lines are plotted as open circles. All *p *values were computed using Welch's *t *test.

More importantly, this query also identified experiments in which estrogen signaling was perturbed in a manner other than the direct addition of ER ligands. For example, in series GSE10911 [22], samples of the aromatase-overexpressing cell line MCF7aro treated with testosterone (which is converted to E2 by aromatase) were positively associated with the E2 treatment signature relative to untreated controls. Beyond identifying experiments using MCF7 cells, several of the series identified by openSESAME were comprised of samples of primary breast tumors or breast cancer cell lines. In these series, ER-positive samples had significantly higher SA scores than ER-negative samples (Figure 3) suggesting that ER-positive tumors and cell lines have higher levels of ER-pathway activation. Finally, openSESAME identified two series in which gene expression was profiled in human endometrial tissue. In series GSE6364 [23] and GSE4888 [24], samples obtained during the proliferative phase of the menstrual cycle had significantly higher SA scores than those obtained during the mid secretory phase.

### openSESAME identifies experiments related to stratified squamous epithelia in GEO using a signature of p63 silencing

The transcription factor p63 is required for the differentiation and maintenance of stratified epithelium. Homozygous p63 knockout mice are born with profound developmental defects, including a lack of epidermal stratification and the absence of all squamous epithelia [25,26], and mutations in p63 are associated with several ectodermal dysplastic syndromes in humans [27]. We used a signature of genes that are differentially expressed following the silencing of p63 in squamous and keratinocytic cell lines [28] (Additional File 3) to identify other conditions in which p63 activity might be modulated (Additional File 4).

**Figure 4:**
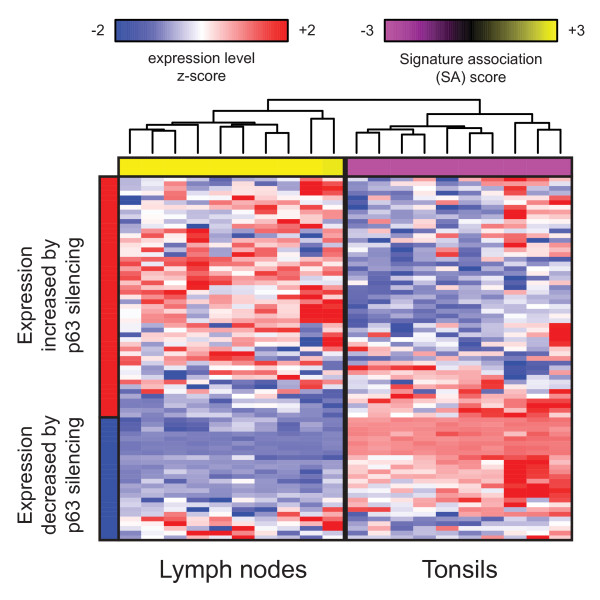
**A gene expression signature of p63 silencing discriminates between lymph nodes and tonsils**. In GEO series GSE2665, openSESAME identified that samples of lymph nodes and tonsillar tissue are positively and negatively associated with the p63-silencing signature, respectively, suggesting that p63 is important for maintenance of the stratified squamous epithelium that is present in tonsils but is absent from lymph nodes.

In many of the series with significant enrichment, the genes in the p63-silencing signature were coordinately differentially expressed between stratified squamous epithelia and other tissue types. For example, in GSE7307 and GSE3526 [29], two series in which dozens of distinct tissue types were transcriptionally profiled, the p63-silencing signature was positively associated with samples of non-stratified epithelia (e.g., endometrium) or non-epithelial tissues (e.g., brain) relative to samples of stratified squamous epithelium (e.g., skin). This was also true in the tumor collection series GSE2109, in which the p63-silencing signature was positively associated with primary tumors of non-stratified epithelial tissues (e.g., breast) relative to tumors arising from stratified squamous epithelium (e.g., esophagus) or squamous cell carcinoma of the lung. Perhaps the most striking example of tissue-specific association with this signature, however, is series GSE2665 [30] (Figure 4). In this series, lymph node samples had a strong positive association with the p63-silencing signature relative to samples from tonsils, which, although comprised of lymphoid tissue, are covered by a layer of stratified squamous epithelium.

**Figure 5:**
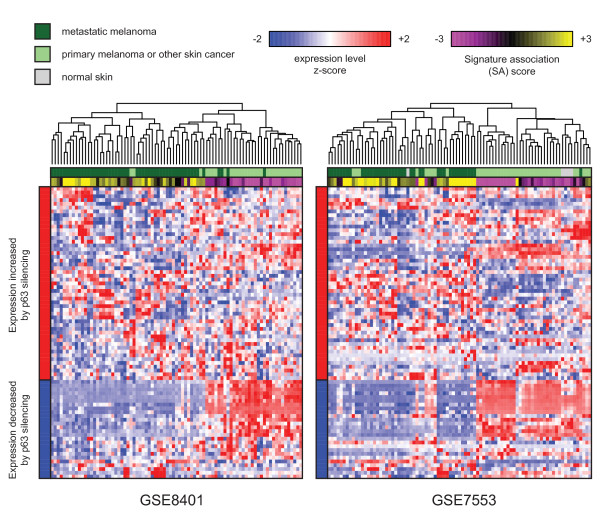
**A gene expression signature of p63 silencing is positively associated with metastatic melanoma**. In two independent GEO series, GSE8401 and GSE7553, openSESAME identified that primary and metastatic melanomas were negatively and positively associated with the p63-silencing signature, respectively.

This query also identified several series pertaining to diseases of stratified epithelial tissue. For example, in series GSE8401 [31] and GSE7553 [32], the gene expression pattern of p63 silencing was reflected in metastatic melanomas, and this pattern was reversed in primary melanoma (Figure 5). In accordance with this apparent decrease in p63 activity in metastatic melanoma, we found that there was a significant decrease in the transcription of the *TP63 *gene itself in these samples (*p *= 2.2 × 10^-10 ^and 4.1 × 10^-3 ^for GSE8401 and GSE7553, respectively, by Student's *t *test; metastatic vs. primary melanoma only).

### Performance of the openSESAME algorithm

We performed several sets of experiments to evaluate the performance of the openSESAME algorithm. We first sought to evaluate the sensitivity and specificity of the Wilcoxon rank-sum test used to compute the SA scores. To do this, we used the original 189-Affymetrix-probeset signature of E2 treatment [2,20] and the publicly available set of ranked fold changes observed between compound-treated MCF7 breast cancer cells and vehicle-treated controls in the Connectivity Map (CMap) [2]. This dataset contains 19 instances of MCF7 cells grown in complete medium treated with E2 (which we considered true positives) and 3076 instances of MCF7 cells grown in complete medium treated with other compounds (which we considered true negatives). We calculated SA scores using the E2 treatment signature for each of these samples and found that the area under the Receiver Operating Characteristics (ROC) curve for distinguishing E2-treated from non-E2-treated samples was 0.981 (Additional File 5 orange curve). For comparison, we used the same E2 treatment signature to calculate S scores (the K-S-test-based statistic used by Lamb et al. [2]) across the same dataset using the Connectivity Map website, and obtained an area under the ROC curve of 0.971 (Additional File 5 purple curve). These results suggest that the openSESAME SA score has similar performance to the CMap S score for distinguishing E2-treated samples from samples treated with other compounds.

We next sought to address how sample size and signature size impact the sensitivity and specificity of the openSESAME algorithm to identify significantly enriched datasets. In these analyses we additionally compared the relative performance of Fisher's exact test and the K-S test to identify significant SA score distributions. For these analyses we chose two GEO series: GSE2225 [33] (a dataset of 18 samples in which MCF7 cells overexpressing aromatase were treated with estrogen, the E2 precursor testosterone, or vehicle) as an example of a dataset that strongly reflects the effects of estrogen receptor pathway activation (Fisher *q *= 3.67 × 10^-6^; K-S *q *= 0.00766), and GSE21653 [34] (a dataset of 266 primary breast tumors) as an example of a dataset that more weakly reflects the effects of estrogen receptor pathway activation (Fisher *q *= 0.319; K-S *q *= 0.198).

To assess the sensitivity of the Fisher and K-S tests as a function of sample size, we randomly selected subsets of samples from each dataset (Additional File 6). As the subset size increased, the power to detect coordinate differential expression of the E2 signature increased. However, the sensitivity achieved using Fisher's exact test with series GSE2225 was considerably higher than that achieved using the K-S test, whereas the sensitivity of the K-S test was moderately higher than that of Fisher's exact test in series GSE21653, suggesting that the K-S test may have utility in identifying weaker patterns of coordinate differential expression. Using either randomly permuted data or normally distributed random data we found that the false positive (Type I) error rate of both methods was well controlled. The specificity of both methods decreased as the sample size increased, and was higher with Fisher's exact test, but the frequency of Type I errors remained below or close to the specified Type I error threshold at all sample sizes (see tables in Additional File 6).

To explore how the performance of openSESAME varies with different types of query signatures, we tested the effect of varying the size or composition of the E2 treatment signature upon its ability to detect the estrogen receptor pathway activation reflected in GSE2225 and GSE21653. The E2 treatment signature contains 111 genes, of which 34 (31%) are up-regulated by E2 treatment. We first examined the effect of varying the size of the signature while maintaining the original proportion of up- and down-regulated genes (Additional File 7). We found that the ability of the openSESAME algorithm to detect perturbation of estrogen signaling increased with the size of the signature. The specificity of the algorithm also varied according to the dataset and the test used, with the greatest deviation from the expected Type I error rate observed in series GSE21653 when the K-S test was used. However, the specificity was very high in both datasets when Fisher's exact test was used.

We next examined how the relative proportion of up- and down-regulated genes in the signature affects the performance of openSESAME (Additional File 8). We used subsets of the E2 treatment signature that contained a total of 68 genes but which varied in the proportion of E2-upregulated genes (from 2-34 genes, or 3% to 50%). The sensitivity was strongly dependent on the relative proportion of up- and down-regulated genes and was highest when the signature was most balanced, i.e., when the number of "up" and "down" genes was equal. Specificity was well maintained regardless of the relative proportion of up- and down-regulated genes.

### Comparison of openSESAME with existing approaches

To further examine the performance of openSESAME, we compared the results of the openSESAME queries performed with the E2 and p63 signatures with the results of similar queries performed using GeneChaser [12] and MARQ [14], two web-based applications that identify experimental comparisons that are relevant to an expression signature of interest. We first translated the expression signatures into gene symbols and attempted to use them in a GeneChaser query. However, although the application correctly recognized these symbols, no results were returned. This appears to be due to a strict requirement of GeneChaser that the expression of all up- or down--regulated genes changes in the same direction between two experimental groups.

We next used the Entrez Gene identifiers of the genes in each expression signature to initiate MARQ queries and compared the results with those of the corresponding openSESAME queries (Additional Files 9 and 10). There were 208 unique GEO datasets that overlapped between the openSESAME and MARQ analyses. Of these datasets, MARQ identified 45 phenotypic comparisons (from 23 unique datasets) that result in a pattern of differential expression of the E2 signature genes that is significantly similar to the E2 signature (FDR-corrected *p *< 0.05) (Additional File 9). Of these 23 datasets, openSESAME recovered four (GSE11352, GSE2225, GSE6364, and GSE4888) and assigned nominal significance to a fifth (GSE7765; Fisher *p *= 0.033, Fisher *q *= 0.84) (Additional File 9). However, it is unclear whether any of the remaining 18 datasets identified by MARQ are also biologically relevant to estrogen signaling (i.e., true positives), and it is therefore challenging to use the overlap between the MARQ and openSESAME results to determine the sensitivity and specificity of openSESAME.

MARQ identified 52 phenotypic comparisons (from 23 unique datasets) that result in a pattern of differential expression of the p63 silencing signature that is significantly similar to the p63 signature (FDR-corrected *p *< 0.05) (Additional File 10). Of these 23 datasets, 9 (GSE2665, GSE6932, GSE1420, GSE3524, GSE7216, GSE6475, GSE2144, GSE6281, and GSE10433) appeared to be directly related to tissues, diseases, or a phenotype of squamous stratified epithelia, but only three of these datasets were also recovered by openSESAME (Additional File 10). The failure of openSESAME to identify six of the nine datasets identified by MARQ where differences in p63 signaling seem biologically plausible suggests that methods like MARQ which leverage phenotypic data for identifying patterns of coordinate differential gene expression may generally be more powerful than phenotype-naïve approaches such as openSESAME, at the cost of requiring that all potentially biologically important phenotypic comparisons are pre-specified.

However, openSESAME was able to detect significant coordinate differential expression of the p63 signature in two series that were both missed by MARQ. For example, GSE6710 [35], a dataset that compared gene expression in psoriatic lesions with normal skin, was assigned a significant *q *value by openSESAME (*q *= 0.0109 by Fisher's exact test), but received only a moderately significant FDR-corrected *p *value (0.0775) from MARQ. openSESAME also assigned high significance (Fisher *q *= 0.000452) to GSE2280 [36], which was not assigned a significant *p *value by MARQ. Within this series, metastatic and non-metastatic oral squamous carcinomas were profiled, and metastatic and non-metastatic samples were usually assigned positive and negative SA scores, respectively. However, it appears that because there was significant heterogeneity between samples from different patients, the comparison performed by MARQ between these two phenotypes did not receive a significant *p *value (FDR-corrected *p *= 0.41). This suggests that openSESAME may be more powerful than methods such as MARQ in detecting coordinate differential expression of a signature in datasets with high variability, such as those comprised of clinical samples.

## Discussion

Databases of genome-wide gene expression data represent a rich source of detailed information about patterns of gene expression that result from experimental perturbations and disease states. An important use of such storehouses is to understand the similarities and differences between various biological perturbations. Current approaches for querying these gene expression databases limit the analysis to comparisons of gene expression differences between pre-determined experimental groups. In contrast, openSESAME searches for instances of coordinate differential gene expression of a query signature without using phenotypic data.

The phenotype-naïve approach of openSESAME is similar in some regards to a strategy developed by West and colleagues [37,38]. In this approach, the major principal components of a gene expression signature identified in the query dataset are used to train a binary regression model to serve as a predictor of signature "activation". This model can then be used to predict signature activation in other datasets and to examine the potential association between signature activation probabilities and phenotypes within these target datasets. For example, probabilities for a gene expression signature reflecting the response to lactic acidosis is associated with better survival in breast cancer patients, consistent with the hypothesis that tumors in these patients may repress glycolysis via inhibition of the Akt pathway [39]. Such signature activation probabilities are thus an alternative to the SA scores calculated by openSESAME in that both can be calculated per sample without regard to phenotypic comparisons. However, we are not aware of work to assess the significance of observed signature activation probabilities across datasets other than by association with phenotypic variables. The aim of this work is to demonstrate the ability of a phenotype-naïve method to identify datasets that are biologically related to each other.

The openSESAME algorithm consists of two components: a signature association (SA) score that reflects the similarity of a sample's pattern of relative gene expression to a query signature, and a test to determine whether the distribution of SA scores of all samples in a dataset represents a significant pattern of coordinate differential expression of the query signature. The SA score is a normalized Wilcoxon rank-sum statistic that compares the expression levels of the up- and down-regulated genes in the query signature. We chose this statistic over parametric methods such as a Pearson correlation to the query signature vector or Student's *t *test to compare the expression levels of the "up" and "down" genes because we could not assume that the normalized expression values would be normally distributed in the absence of significant coordinate differential expression. Using a signature of E2 treatment and a dataset of MCF7 cells treated with various compounds, including E2, we have validated that this SA score is a sensitive and specific method for detecting differential expression of a query signature.

We have explored two approaches to determine whether the SA scores for all samples in a dataset represents significant coordinate differential expression of a query signature. One method first establishes the significance of each SA score using threshold values and then uses Fisher's exact test to determine whether the proportion of samples exceeding these thresholds in each dataset is significantly different from the proportion of samples exceeding these thresholds across all samples in the compendium. The threshold values are determined based on the FDR corrected *p *value (*q *value) of the SA scores. The second method uses a Kolmogorov-Smirnov (K-S) test to determine whether the distribution of SA scores in each dataset is significantly different from the distribution of all SA scores across all samples in the compendium. The specificity of Fisher's exact test appears to be superior to that of the K-S test, presumably because the categorical assignment of significance to SA scores acts as a "band-pass filter" to remove noise from moderately skewed distributions of SA scores. However, the relative sensitivity of the Fisher's exact and K-S tests seems to be dependent on the strength of the coordinate differential signature expression signal, the former being more sensitive when the signal is strong and the latter being more sensitive when the signal is weaker.

To explore how openSESAME might perform with other query signatures, we examined how the performance of openSESAME depends on the size of the query signature and the relative proportion of up- and down-regulated genes. To do this, we sampled genes from the E2 treatment signature and tested the ability of these derivative queries to detect significant estrogen pathway activation in two datasets: one in which the gene expression signal of estrogen pathway activation was strong (GSE2225 [33]) and another in which the signal is more difficult to detect (GSE21653 [34]). These analyses indicate that the performance of openSESAME increases as both the size of the query signature increases and the relative proportion of up- and down- regulated genes becomes more balanced, although the absolute performance of openSESAME is strongly dependent upon the strength of the signal of coordinate differential expression. For example, when using Fisher's exact test, openSESAME achieves > 90% sensitivity to detect the estrogen pathway activation in GSE2225 at a *p *value threshold of 0.001 when the signature contains at least 80 genes and 30% of the signature is composed of upregulated genes. Similar performance is achieved with a 68-gene signature containing 47% upregulated genes. However, in GSE21653, which has a weaker signal of estrogen pathway activation, the overall effect of signature size and composition upon performance remains similar, but openSESAME has only 11% sensitivity to detect the activation of the estrogen pathway at *p *< 0.001 using Fisher's exact test (although sensitivity increases 23% using the K-S test). These analyses suggest the potential utility of openSESAME to identify coordinate differential expression signals with a wide range of signature sizes of varying composition.

We used a similar approach to explore how the size of a target dataset affects the ability of openSESAME to detect coordinate differential expression of a query signature within that dataset. The power of openSESAME to detect coordinate differential expression of a query signature generally increased with the size of the target dataset, but the absolute performance of the algorithm was again highly dependent upon the strength of the signal of coordinate differential expression. In series GSE2225, which has a strong estrogen pathway activation signal, openSESAME achieves > 90% sensitivity to detect this signal in subsets of GSE2225 as small as 12 samples (67% of the full-size dataset) at a *p *value threshold of 0.001 using Fisher's exact test. However, in GSE21653, which has a weaker signal of estrogen pathway activation, openSESAME only achieves > 90% sensitivity to detect this signal in subsets of GSE21653 containing between 225 and 250 samples (85-94% of the full-size dataset) at a *p *value threshold of 0.01 using the K-S test. With approximately the same number of samples, Fisher's exact test only achieves 36% sensitivity to detect the pathway activation signal at the same *p *value threshold. These analyses suggest the potential for openSESAME to detect strong signals of coordinate differential gene expression in small datasets and the potential importance of the K-S approach and larger datasets to powerfully detect weaker signals of coordinate differential gene expression.

We used openSESAME to identify experiments related to estrogen receptor (ER) pathway activation in a collection of approximately 75,000 human samples profiled on Affymetrix microarrays that have been deposited in the Gene Expression Omnibus (GEO) data repository. Our success in identifying numerous datasets representing differential ER pathway activation is due not only to the size of the gene expression compendium we searched, but also the relatively high frequency with which the ER pathway is modulated in the experimental and clinical datasets contained within this repository due to its biological and clinical importance. We identified experiments related to ER pathway activation using a signature of genes that are differentially expressed in response to E2 treatment in MCF7 cells. openSESAME identified experiments in which MCF7 cells were perturbed with E2, phytoestrogens such as genistein, or the E2 precursor testosterone (in cells overexpressing aromatase).

In addition to these results, we identified significant enrichment of the estrogen pathway activation signature in several series of primary breast tumors or breast cancer cell lines and found that ER-positive tumors and cell lines had significantly higher association with the E2 treatment signature than did ER-negative samples. These results likely reflect increases in steady-state ER pathway activation in response to available estrogen in ER-positive tumors and cell lines. Similarly, openSESAME identified two series of human endometrial tissue samples in which the E2 treatment signature was positively associated with the proliferative phase of the menstrual cycle and negatively associated with the mid secretory phase. These two phases correspond to higher and lower serum estrogen levels, respectively, suggesting that the perturbation of the E2 treatment signature in these datasets reflects a response to changing levels of available estrogen.

We also explored conditions related to the differentiation and maintenance of stratified squamous epithelia using a gene expression signature of the consequences of p63 downregulation. In two independent experiments, the pattern of gene expression associated with p63 silencing was evident in samples of metastatic melanomas relative to primary melanomas. Subsequent analysis of these datasets confirmed that the expression of *TP63 *is down regulated in metastatic melanoma. This confirms previously published observations in which p63 staining was absent from greater than 90% of malignant melanomas [40-44] and may offer a mechanistic explanation as to why genes involved in stratified epithelial differentiation are markedly downregulated in such tumors [32]. Taken together, the results of this openSESAME query implicate p63 as a regulator of the differentiation, maintenance and proliferation of stratified epithelium, highlighting the utility of openSESAME for the elucidation of gene function.

Finally, we chose to compare openSESAME with two other methods that relate gene expression differences in GEO-deposited data to a query signature. One of the key differences between openSESAME and both GeneChaser [12] and MARQ [14] is that, unlike openSESAME, these methods precompute the degree of differential expression associated with phenotypic comparisons gleaned from sample annotation information that has been deposited together with the gene expression data. We found that GeneChaser was not useful if any of the genes in the "up" or "down" sets were not differentially expressed in the same direction as the others in each set. This highlights one of the strengths of openSESAME, which is that it can identify experiments that are relevant to a signature of interest even if a subset of genes in a signature are not differentially expressed in the same direction in two different experiments.

Using the E2 treatment signature, MARQ identified perturbations of estrogen signaling in four of the series detected by openSESAME. Similarly, when using the p63 silencing signature, MARQ identified phenotypic comparisons related to stratified squamous cell type or differentiation within three of the series detected by openSESAME. MARQ also identified phenotypic comparisons within a number of series that were not detected by openSESAME. For example, MARQ identified E2 signaling as downregulated in MCF7 cells treated with dioxin, which is a well-known antiestrogenic agent [45], and identified p63 signaling as being upregulated in acne lesions compared with normal skin and in the bronchial epithelium of smokers compared with that of nonsmokers (both of which may reflect squamous repair mechanisms in injured or inflamed tissue). The failure of openSESAME to identify datasets identified by MARQ suggests that methods like MARQ that leverage phenotypic data to identify patterns of coordinate differential gene expression may generally be more sensitive than phenotype-naïve approaches such as openSESAME, as they incorporate additional sources of information.

However, there were a number of datasets that were identified by openSESAME that were not identified by MARQ, and these point to potential advantages of a phenotype-naïve approach. For example, openSESAME detected significant perturbation of the p63 silencing signature in GSE2280 [36], in which p63 silencing is positively associated with metastatic squamous cancer samples, consistent with the loss of p63 expression observed in the metastatic melanoma samples described earlier. MARQ did not assign a significant *p *value to this comparison, however, because there was substantial heterogeneity among these samples. Taken together, these results suggest that methods such as MARQ may generally be more specific in identifying interesting biological perturbations, given the directed nature of its queries, but openSESAME is more robust to variability within prespecified comparisons. There were also a number of examples of significant coordinate differential expression of the E2 and p63 query signatures that were detected by openSESAME but were not detected by MARQ (Additional Files 2 and 4), including the phytoestrogen and melanoma datasets, because these datasets were not included in the curated data from which MARQ calculated phenotype-associated differential expression. The perturbations that are detected uniquely by openSESAME highlight the key advantage of openSESAME in that it is able to detect coordinate differential expression of a query signature without regard to explicit annotation-driven comparisons between defined experimental groups.

In this work, we have shown that openSESAME can identify datasets with similar patterns of coordinate differential gene expression using an approach that does not require identification of phenotype-associated differential expression and that these gene expression similarities can be used to identify conditions that are biologically related to each other. We therefore believe that openSESAME will be a broadly useful approach to leverage the increasing body of available genome-wide gene expression profiling experiments to generate hypotheses about the causes and consequences of observed patterns of differential gene expression. To further explore the potential of this approach, we have created a publicly accessible implementation of openSESAME (http://opensesame.bu.edu) for the scientific community to test with their own gene expression signatures.

## Conclusions

We have shown that openSESAME can identify datasets with similar patterns of coordinate differential gene expression in the absence of explicit phenotypic comparisons and that these similarities can be used to identify conditions that are biologically related to each other. openSESAME represents a novel approach to leverage the increasing body of available genome-wide gene expression profiling experiments to generate hypotheses about the causes and consequences of observed patterns of differential gene expression. To encourage the community to explore the potential utility of this approach, we have created a publicly accessible implementation of openSESAME (http://opensesame.bu.edu) for the scientific community to test with their own gene expression signatures.

## Methods

### Software and Hardware

All computations were performed in the R programming environment (version 2.11.1). The R packages affy [46] and limma [47] were obtained from Bioconductor (http://www.bioconductor.org) and the package multtest [48] was obtained from the Comprehensive R Archive Network (CRAN) (http://cran.r-project.org). Entrez Gene ID-specific probeset mappings for Affymetrix arrays (version 13.0.0) [49] were obtained from the Molecular and Behavioral Neuroscience Institute at the University of Michigan (http://brainarray.mbni.med.umich.edu/Brainarray/Database/CustomCDF). All computations were performed using the Linux cluster for Genetic Analysis (LinGA) at the Boston University Medical Campus.

### Gene Expression Omnibus (GEO) dataset acquisition and normalization

All publicly available CEL files from the Gene Expression Omnibus data repository [50] as of September 23, 2010 in which RNA had been profiled on an HG-U133 generation (HG-U133A, HG-U133B, HT-HG-U133A, HG-U133A 2.0, or HG-U133 Plus 2.0) Affymetrix GeneChip were downloaded. This represented 2183 submissions to GEO of 75164 samples in total. Expression estimates for each sample within each series were derived using the Robust Multichip Average (RMA) [51] (Figure 1a) using version 13.0.0 BrainArray Entrez Gene ID-specific CDFs. These data were then *z*-score normalized (to a mean of zero and standard deviation of one) (Figure 1b) by microarray platform within each GEO "series" submission (a collection of array samples that have been deposited as a group, often in connection with a manuscript) (Figure 1c).

### Computing signature association (SA) scores between microarray samples and an expression signature

For each sample, the relative expression values of the signature genes (calculated relative to the mean of the samples in the dataset) are ranked and a Wilcoxon rank-sum test is then performed to determine whether the ranked expression levels of the genes from the "up" and "down" sets are equally distributed (Figure 1d). The Wilcoxon rank-sum test computes a *W *statistic from the ranks *r_up _*of the "up" genes and from the sizes of the "up" and "down" gene sets (*n_up _*and *n_down_*, respectively):

$$W=\sum r_{up}-\frac{n_{up}\left(n_{up}+1\right)}{2}$$

The *W *statistic is then normalized to the mean μ and standard deviation σ of the null distribution of *W *to generate a statistic termed a *signature association (SA) score:*

$$\mu =\frac{\left(n_{up}\cdot n_{down}\right)}{2}$$

$$\sigma =\frac{\left(n_{up}\cdot n_{down}\right)\left(n_{up}+n_{down}+1\right)}{12}$$

$$SA=\frac{\left(W-\mu \right)}{\sigma }$$

The distribution of the SA score approximates a *t *distribution with (*n_up_*+*n_down_*-2) degrees of freedom. Positive SA scores indicate that the relative expression values of the "up" genes in the signature are ranked more highly than the "down" genes in that sample, whereas negative SA scores indicate that the "down" genes are ranked more highly than the "up" genes. SA scores close to zero indicate that in that sample the signature genes are not coordinately induced or repressed in the same pattern as defined by the signature.

### Identifying datasets that are significantly enriched in samples associated with an expression signature

In the setting of expression levels that are determined relative to the mean expression across a group of related samples (e.g., a GEO series), the sign of each sample's SA score indicates whether the "up" and "down" genes in the signature are coordinately induced or repressed in that sample relative to the mean of all samples in that group. To determine whether the distribution of SA scores in a particular dataset represents significant coordinate perturbation of the expression of the signature genes, a two-sided Kolmogorov-Smirnov (K-S) test is performed to determine whether the observed distribution of SA scores differs from the distribution of SA scores across all samples in all datasets (Figure 1e, upper panels). Alternately, the significance of each sample's SA score is evaluated using an FDR-corrected *p *value (*q *value) cutoff of 0.1, and Fisher's exact test is used to determine whether the categorical distribution of SA scores in a given dataset (significantly positive, significantly negative, or not significantly changed) is significantly different from the categorical distribution of SA scores across all samples in all datasets (Figure 1e, lower panels). In addition, the *p *value of the Fisher's exact test for any dataset with a *greater *proportion of non-significant SA scores than the background distribution is set to 1. Correction for multiple hypothesis testing was accomplished using the Benjamini-Hochberg false discovery rate (FDR) [52].

### Expression signatures

A list of genes whose expression was induced or repressed greater than 2.5-fold in MCF7 cells treated for 8 or 48 hours with 10 nM 17-β-estradiol (E2) (relative to vehicle-treated controls) was previously reported [20]. The Entrez Gene identifiers that correspond to these gene symbols were identified, and 34 upregulated and 77 downregulated identifiers were used as a signature of differential gene expression in response to E2 treatment (Additional File 1). Another report [28] contained a list of genes whose expression was induced or repressed greater than 2-fold in at least three of five cell lines (two keratinocytic lines and three squamous carcinoma lines) transformed with a vector expressing a p63-specific shRNA (relative to the same cell lines transformed with a control vector expressing an shRNA targeting GFP). This list was used to derive a p63-silencing signature of 51 upregulated and 26 downregulated Entrez Gene identifiers (Additional File 4).

## Authors' contributions

The openSESAME method was originally conceived and implemented by MEL and was further developed and improved by ACG. All analyses were performed by ACG. ACG, AS and MEL wrote the manuscript. All authors read and approved the final manuscript.

## Supplementary Material

Additional file 1 **A gene expression signature of E2 treatment**. The expression of these 111 genes was previously reported to be increased or decreased > 2.5-fold by the treatment of MCF7 cells with 10 nM 17-β-estradiol (E2) for either 8 or 48 hours (or both) relative to treatment with vehicle[20]. Click here for file

Additional file 2 **Results of openSESAME query using E2 treatment signature**. In an openSESAME query using the signature of E2 treatment, these 56 GEO series were assigned an FDR *q *< 0.25 with either the K-S or Fisher's exact tests. Series are sorted in ascending order by K-S *q *value. Click here for file

Additional file 3 **A gene expression signature of p63 silencing**. The expression of these 77 genes was previously reported to be increased or decreased > 2-fold in at least three of five cell lines treated with an shRNA targeting p63, relative to the same cell lines treated with an shRNA targeting GFP[28]. Click here for file

Additional file 4 **Results of openSESAME query using p63 silencing signature**. In an openSESAME query using the signature of p63 silencing, these 123 GEO series were assigned an FDR *q *< 0.25 with either the K-S or Fisher's exact tests. Series are sorted in ascending order by K-S *q *value. Click here for file

Additional file 5 **Receiver Operating Curves (ROCs) using E2 treatment signature in Connectivity Map (CMap) MCF7 dataset**. An openSESAME query was performed using the original 189-Affymetrix-probeset E2 treatment signature on the ranked fold changes from all instances of treatment of MCF7 cells in the CMap build 2.0 dataset. A ROC was constructed (orange) in which instances of treatment of MCF7 with 17-β-estradiol (E2) were considered true positives. Another ROC was constructed (purple) using the S scores from a web query of the Connectivity Map using the same signature. Click here for file

Additional file 6 **Variation of openSESAME *p *values with sample size in GEO series GSE2225 and GSE21653**. **A, B**. For each sample size, 1000 subsets of each GEO series were obtained by permutation and SA scores were computed. Fisher's exact test or a two-sided Kolmogorov-Smirnov (K-S) test were used to compute *p *values for each permutation. **C, D**. The expression values of each gene were shuffled independently 100 times, and for each shuffled dataset, 10 subsets were obtained for each sample size and SA scores and *p *values were computed. **E, F**. A total of 100 simulated datasets were obtained by generating random values from a standard normal distribution and z-normalizing each row ("gene") across all columns ("samples"). For each simulated dataset, 10 subsets were obtained for each sample size and SA scores and *p *values were computed. Below all panels, the fraction of permutations with *p *values below each threshold is shown. Click here for file

Additional file 7 **Variation of openSESAME *p *values with signature size in GEO series GSE2225 and GSE21653**. **A, B**. For each subset size, 1000 subsets of the signature genes were obtained by permutation, maintaining the same proportion of up- and down-regulated genes in the original signature, and SA scores were computed using each GEO series. Fisher's exact test or a two-sided Kolmogorov-Smirnov (K-S) test were used to compute *p *values for each permutation. **C, D**. The expression values of each gene were shuffled independently 100 times, and for each shuffled dataset, 10 subsets of the signature genes were obtained for each subset size, and SA scores and *p *values were computed. **E, F**. A total of 100 simulated datasets were obtained by generating random values from a standard normal distribution and z-normalizing each row ("gene") across all columns ("samples"). For each simulated dataset, 10 subsets of the signature genes were obtained for each subset size and SA scores and *p *values were computed. Click here for file

Additional file 8 **Variation of openSESAME *p *values with signature composition in GEO series GSE2225 and GSE21653**. **A, B**. For each subset size, 1000 subsets of the up-and down-regulated genes in the signature were obtained by permutation, maintaining a constant signature size, and SA scores were computed using each GEO series. Fisher's exact test or a two-sided Kolmogorov-Smirnov (K-S) test were used to compute *p *values for each permutation. **C, D**. The expression values of each gene were shuffled independently 100 times, and for each shuffled dataset, 10 subsets of the up- and down-regulated genes were obtained for each subset size and SA scores and *p *values were computed. **E, F**. A total of 100 simulated datasets were obtained by generating random values from a standard normal distribution and z-normalizing each row ("gene") across all columns ("samples"). For each simulated dataset, 10 subsets of the up- and down-regulated genes were obtained for each subset size and SA scores and *p *values were computed. Click here for file

Additional file 9 **Comparison of MARQ and openSESAME query results using the E2 treatment signature**. In queries using the E2 treatment signature, these GEO series were assigned an FDR *q *< 0.05 by openSESAME with either the K-S or Fisher's exact tests or contained an experimental comparison to which MARQ assigned an FDR-corrected *p *< 0.05. Series are sorted in ascending order by Fisher q value. Click here for file

Additional file 10 **Comparison of MARQ and openSESAME query results using the p63 silencing signature**. In queries using the p63 silencing signature, these GEO series were assigned an FDR *q *< 0.05 by openSESAME with either the K-S or Fisher's exact tests or contained an experimental comparison to which MARQ assigned an FDR-corrected *p *< 0.05. Series are sorted in ascending order by Fisher *q *value. Click here for file
