# Supplementary material for: Discovering biological connections between experimental conditions based on common patterns of differential gene expression
Source: BMC Bioinformatics. 2011 Sep 27;12:381. doi: 10.1186/1471-2105-12-381 (PMC3203354; doi:10.1186/1471-2105-12-381)
Supplement: Additional file 5 — Receiver Operating Curves (ROCs) using E2 treatment signature in Connectivity Map (CMap) MCF7 dataset. An openSESAME query was performed using the original 189-Affymetrix-probeset E2 treatment signature on the ranked fold changes from all instances of treatment of MCF7 cells in the CMap build 2.0 dataset. A ROC was constructed (orange) in which instances of treatment of MCF7 with 17-β-estradiol (E2) were considered true positives. Another ROC was constructed (purple) using the S scores from a web query of the Connectivity Map using the same signature. [file 1471-2105-12-381-S5.PDF]

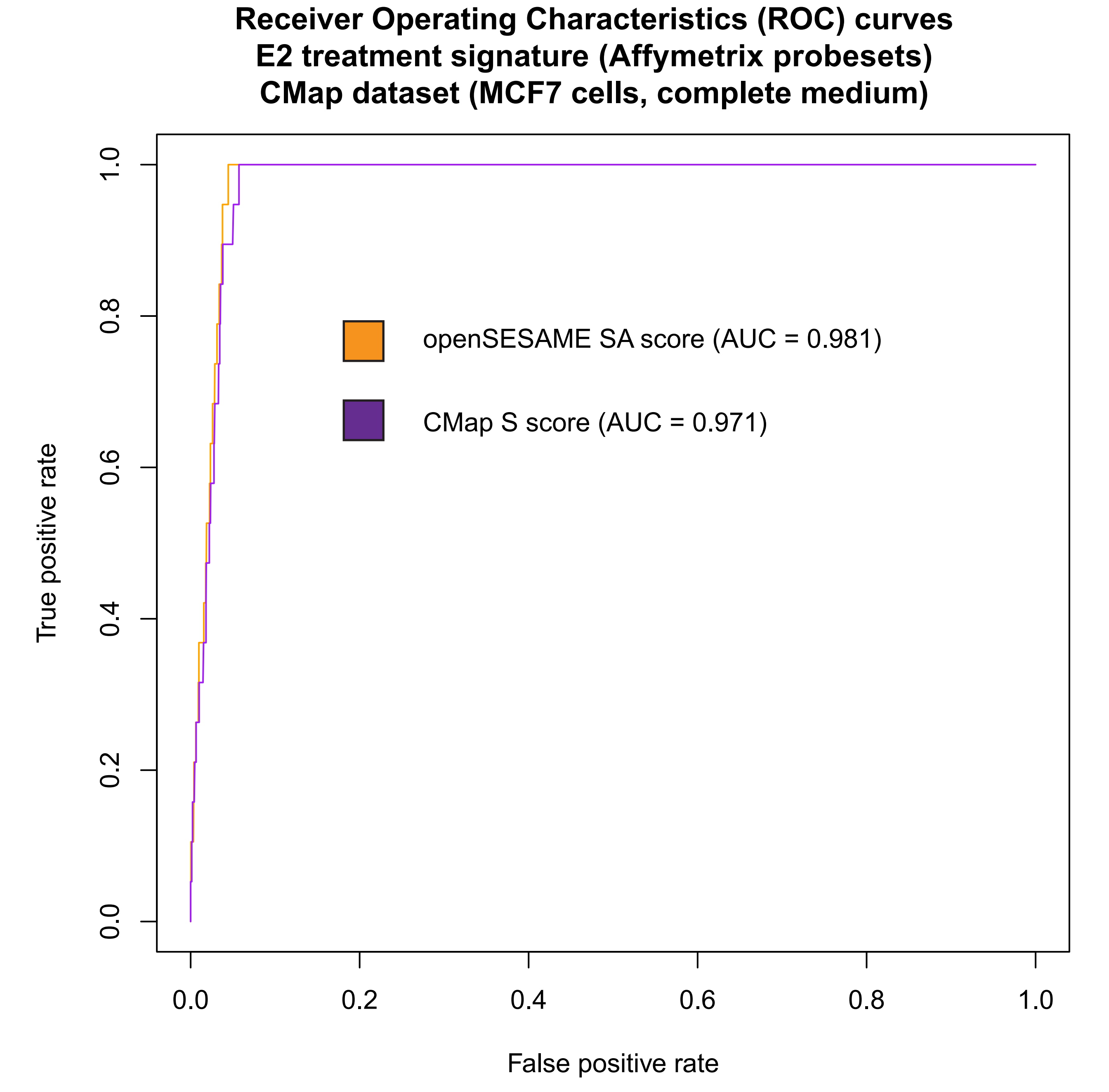

Receiver Operating Characteristics (ROC) curves
E2 treatment signature (Affymetrix probesets)
CMap dataset (MCF7 cells, complete medium)
openSESAME SA score (AUC = 0.981)
CMap S score (AUC = 0.971)
True positive rate
False positive rate
0.0
0.2
0.4
0.6
0.8
1.0
