# Supplementary material for: Discovering biological connections between experimental conditions based on common patterns of differential gene expression
Source: BMC Bioinformatics. 2011 Sep 27;12:381. doi: 10.1186/1471-2105-12-381 (PMC3203354; doi:10.1186/1471-2105-12-381)
Supplement: Additional file 6 — Variation of openSESAME p values with sample size in GEO series GSE2225 and GSE21653. A, B. For each sample size, 1000 subsets of each GEO series were obtained by permutation and SA scores were computed. Fisher's exact test or a two-sided Kolmogorov-Smirnov (K-S) test were used to compute p values for each permutation. C, D. The expression values of each gene were shuffled independently 100 times, and for each shuffled dataset, 10 subsets were obtained for each sample size and SA scores and p values were computed. E, F. A total of 100 simulated datasets were obtained by generating random values from a standard normal distribution and z-normalizing each row ("gene") across all columns ("samples"). For each simulated dataset, 10 subsets were obtained for each sample size and SA scores and p values were computed. Below all panels, the fraction of permutations with p values below each threshold is shown. [file 1471-2105-12-381-S6.PDF]

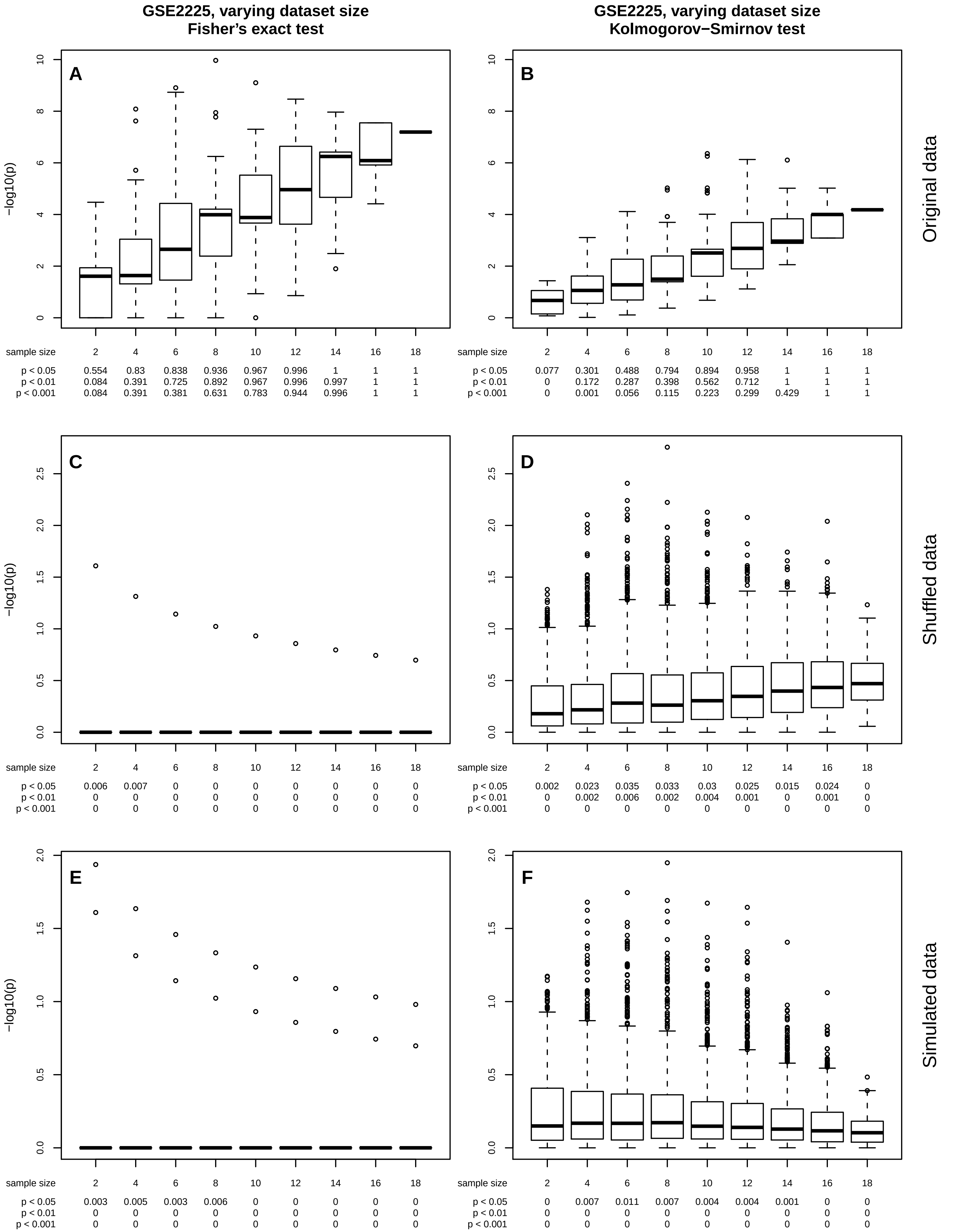

GSE2225, varying dataset size
Fisher's exact test
GSE2225, varying dataset size
Kolmogorov−Smirnov test
A
B
C
D
E
F
−log10(p)
Original data
Shuffled data
Simulated data
sample size 2 4 6 8 10 12 14 16 18
p < 0.05 0.554 0.83 0.838 0.936 0.967 0.996 1 1 1
p < 0.01 0.084 0.391 0.725 0.892 0.967 0.996 0.997 1 1
p < 0.001 0.084 0.391 0.381 0.631 0.783 0.944 0.996 1 1
sample size 2 4 6 8 10 12 14 16 18
p < 0.05 0.077 0.301 0.488 0.794 0.894 0.958 1 1 1
p < 0.01 0 0.172 0.287 0.398 0.562 0.712 1 1 1
p < 0.001 0 0.001 0.056 0.115 0.223 0.299 0.429 1 1
sample size 2 4 6 8 10 12 14 16 18
p < 0.05 0.006 0.007 0 0 0 0 0 0 0
p < 0.01 0 0 0 0 0 0 0 0 0
p < 0.001 0 0 0 0 0 0 0 0 0
sample size 2 4 6 8 10 12 14 16 18
p < 0.05 0.002 0.023 0.035 0.033 0.03 0.025 0.015 0.024 0
p < 0.01 0 0.002 0.006 0.002 0.004 0.001 0 0.001 0
p < 0.001 0 0 0 0 0 0 0 0 0
sample size 2 4 6 8 10 12 14 16 18
p < 0.05 0.003 0.005 0.003 0.006 0 0 0 0 0
p < 0.01 0 0 0 0 0 0 0 0 0
p < 0.001 0 0 0 0 0 0 0 0 0
sample size 2 4 6 8 10 12 14 16 18
p < 0.05 0 0.007 0.011 0.007 0.004 0.004 0.001 0 0
p < 0.01 0 0 0 0 0 0 0 0 0
p < 0.001 0 0 0 0 0 0 0 0 0

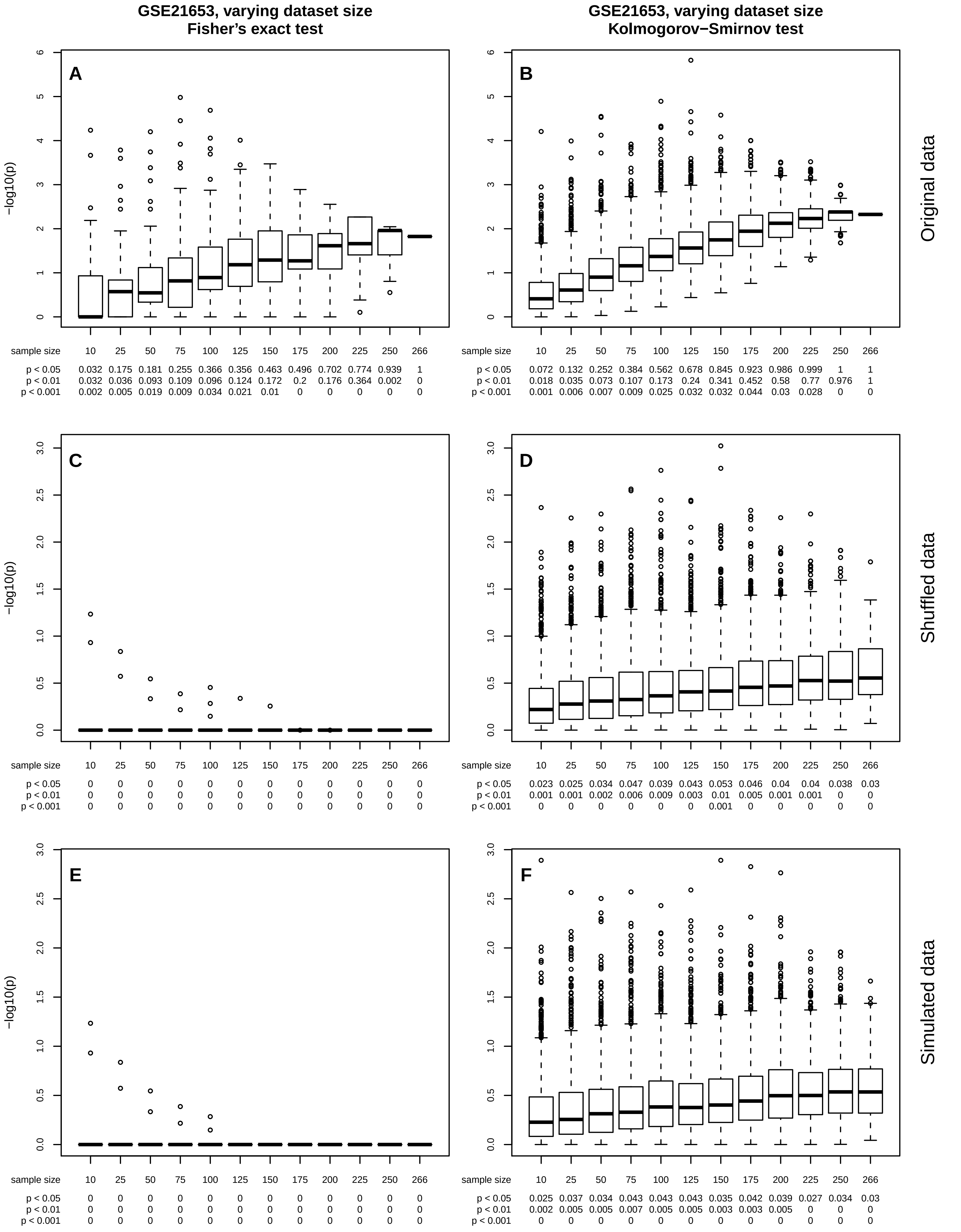
GSE21653, varying dataset size
Fisher's exact test
GSE21653, varying dataset size
Kolmogorov−Smirnov test
A
B
C
D
E
F
−log10(p)
Original data
Shuffled data
Simulated data
sample size 10 25 50 75 100 125 150 175 200 225 250 266
A: p < 0.05 0.032 0.175 0.181 0.255 0.366 0.356 0.463 0.496 0.702 0.774 0.939 1
A: p < 0.01 0.032 0.036 0.093 0.109 0.096 0.124 0.172 0.2 0.176 0.364 0.002 0
A: p < 0.001 0.002 0.005 0.019 0.009 0.034 0.021 0.01 0 0 0 0 0
B: p < 0.05 0.072 0.132 0.252 0.384 0.562 0.678 0.845 0.923 0.986 0.999 1 1
B: p < 0.01 0.018 0.035 0.073 0.107 0.173 0.24 0.341 0.452 0.58 0.77 0.976 1
B: p < 0.001 0.001 0.006 0.007 0.009 0.025 0.032 0.032 0.044 0.03 0.028 0 0
C: p < 0.05 0 0 0 0 0 0 0 0 0 0 0 0
C: p < 0.01 0 0 0 0 0 0 0 0 0 0 0 0
C: p < 0.001 0 0 0 0 0 0 0 0 0 0 0 0
D: p < 0.05 0.023 0.025 0.034 0.047 0.039 0.043 0.053 0.046 0.04 0.04 0.038 0.03
D: p < 0.01 0.001 0.001 0.002 0.006 0.009 0.003 0.01 0.005 0.001 0.001 0 0
D: p < 0.001 0 0 0 0 0 0 0.001 0 0 0 0 0
E: p < 0.05 0 0 0 0 0 0 0 0 0 0 0 0
E: p < 0.01 0 0 0 0 0 0 0 0 0 0 0 0
E: p < 0.001 0 0 0 0 0 0 0 0 0 0 0 0
F: p < 0.05 0.025 0.037 0.034 0.043 0.043 0.043 0.035 0.042 0.039 0.027 0.034 0.03
F: p < 0.01 0.002 0.005 0.005 0.007 0.005 0.005 0.003 0.003 0.005 0 0 0
F: p < 0.001 0 0 0 0 0 0 0 0 0 0 0 0
