# Supplementary material for: Discovering biological connections between experimental conditions based on common patterns of differential gene expression
Source: BMC Bioinformatics. 2011 Sep 27;12:381. doi: 10.1186/1471-2105-12-381 (PMC3203354; doi:10.1186/1471-2105-12-381)
Supplement: Additional file 7 — Variation of openSESAME p values with signature size in GEO series GSE2225 and GSE21653. A, B. For each subset size, 1000 subsets of the signature genes were obtained by permutation, maintaining the same proportion of up- and down-regulated genes in the original signature, and SA scores were computed using each GEO series. Fisher's exact test or a two-sided Kolmogorov-Smirnov (K-S) test were used to compute p values for each permutation. C, D. The expression values of each gene were shuffled independently 100 times, and for each shuffled dataset, 10 subsets of the signature genes were obtained for each subset size, and SA scores and p values were computed. E, F. A total of 100 simulated datasets were obtained by generating random values from a standard normal distribution and z-normalizing each row ("gene") across all columns ("samples"). For each simulated dataset, 10 subsets of the signature genes were obtained for each subset size and SA scores and p values were computed. [file 1471-2105-12-381-S7.PDF]

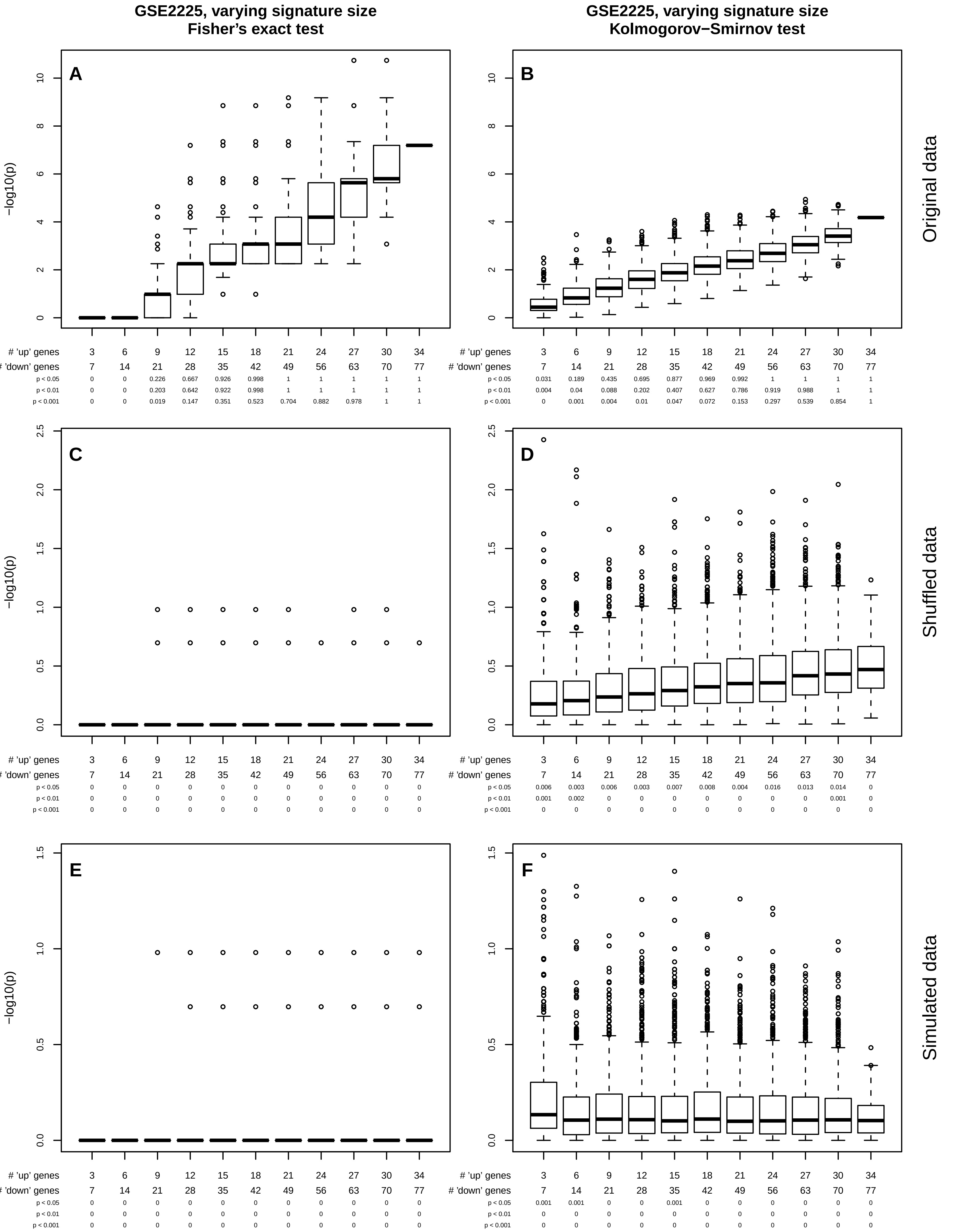
GSE2225, varying signature size
Fisher's exact test
GSE2225, varying signature size
Kolmogorov−Smirnov test
A
B
−log10(p)
Original data
'up' genes 3 6 9 12 15 18 21 24 27 30 34
'down' genes 7 14 21 28 35 42 49 56 63 70 77
p < 0.05 0 0 0.226 0.667 0.926 0.998 1 1 1 1 1
p < 0.01 0 0 0.203 0.642 0.922 0.998 1 1 1 1 1
p < 0.001 0 0 0.019 0.147 0.351 0.523 0.704 0.882 0.978 1 1
'up' genes 3 6 9 12 15 18 21 24 27 30 34
'down' genes 7 14 21 28 35 42 49 56 63 70 77
p < 0.05 0.031 0.189 0.435 0.695 0.877 0.969 0.992 1 1 1 1
p < 0.01 0.004 0.04 0.088 0.202 0.407 0.627 0.786 0.919 0.988 1 1
p < 0.001 0 0.001 0.004 0.01 0.047 0.072 0.153 0.297 0.539 0.854 1
C
D
−log10(p)
Shuffled data
'up' genes 3 6 9 12 15 18 21 24 27 30 34
'down' genes 7 14 21 28 35 42 49 56 63 70 77
p < 0.05 0 0 0 0 0 0 0 0 0 0 0
p < 0.01 0 0 0 0 0 0 0 0 0 0 0
p < 0.001 0 0 0 0 0 0 0 0 0 0 0
'up' genes 3 6 9 12 15 18 21 24 27 30 34
'down' genes 7 14 21 28 35 42 49 56 63 70 77
p < 0.05 0.006 0.003 0.006 0.003 0.007 0.008 0.004 0.016 0.013 0.014 0
p < 0.01 0.001 0.002 0 0 0 0 0 0 0 0.001 0
p < 0.001 0 0 0 0 0 0 0 0 0 0 0
E
F
−log10(p)
Simulated data
'up' genes 3 6 9 12 15 18 21 24 27 30 34
'down' genes 7 14 21 28 35 42 49 56 63 70 77
p < 0.05 0 0 0 0 0 0 0 0 0 0 0
p < 0.01 0 0 0 0 0 0 0 0 0 0 0
p < 0.001 0 0 0 0 0 0 0 0 0 0 0
'up' genes 3 6 9 12 15 18 21 24 27 30 34
'down' genes 7 14 21 28 35 42 49 56 63 70 77
p < 0.05 0.001 0.001 0 0 0.001 0 0 0 0 0 0
p < 0.01 0 0 0 0 0 0 0 0 0 0 0
p < 0.001 0 0 0 0 0 0 0 0 0 0 0

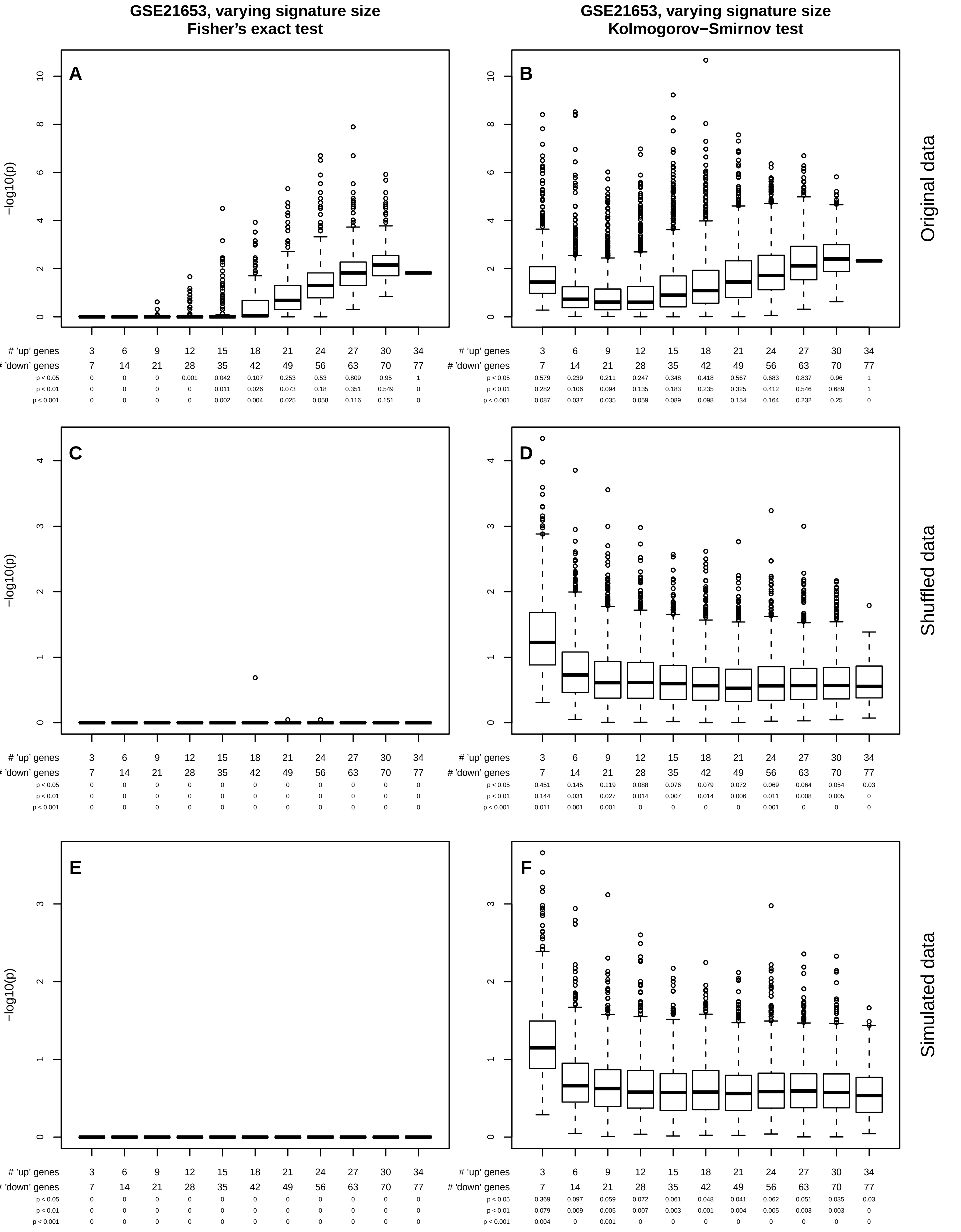
GSE21653, varying signature size
Fisher's exact test
GSE21653, varying signature size
Kolmogorov−Smirnov test
A
B
−log10(p)
Original data
'up' genes 3 6 9 12 15 18 21 24 27 30 34
'down' genes 7 14 21 28 35 42 49 56 63 70 77
p < 0.05 0 0 0 0.001 0.042 0.107 0.253 0.53 0.809 0.95 1
p < 0.01 0 0 0 0 0.011 0.026 0.073 0.18 0.351 0.549 0
p < 0.001 0 0 0 0 0.002 0.004 0.025 0.058 0.116 0.151 0
'up' genes 3 6 9 12 15 18 21 24 27 30 34
'down' genes 7 14 21 28 35 42 49 56 63 70 77
p < 0.05 0.579 0.239 0.211 0.247 0.348 0.418 0.567 0.683 0.837 0.96 1
p < 0.01 0.282 0.106 0.094 0.135 0.183 0.235 0.325 0.412 0.546 0.689 1
p < 0.001 0.087 0.037 0.035 0.059 0.089 0.098 0.134 0.164 0.232 0.25 0
C
D
−log10(p)
Shuffled data
'up' genes 3 6 9 12 15 18 21 24 27 30 34
'down' genes 7 14 21 28 35 42 49 56 63 70 77
p < 0.05 0 0 0 0 0 0 0 0 0 0 0
p < 0.01 0 0 0 0 0 0 0 0 0 0 0
p < 0.001 0 0 0 0 0 0 0 0 0 0 0
'up' genes 3 6 9 12 15 18 21 24 27 30 34
'down' genes 7 14 21 28 35 42 49 56 63 70 77
p < 0.05 0.451 0.145 0.119 0.088 0.076 0.079 0.072 0.069 0.064 0.054 0.03
p < 0.01 0.144 0.031 0.027 0.014 0.007 0.014 0.006 0.011 0.008 0.005 0
p < 0.001 0.011 0.001 0.001 0 0 0 0 0.001 0 0 0
E
F
−log10(p)
Simulated data
'up' genes 3 6 9 12 15 18 21 24 27 30 34
'down' genes 7 14 21 28 35 42 49 56 63 70 77
p < 0.05 0 0 0 0 0 0 0 0 0 0 0
p < 0.01 0 0 0 0 0 0 0 0 0 0 0
p < 0.001 0 0 0 0 0 0 0 0 0 0 0
'up' genes 3 6 9 12 15 18 21 24 27 30 34
'down' genes 7 14 21 28 35 42 49 56 63 70 77
p < 0.05 0.369 0.097 0.059 0.072 0.061 0.048 0.041 0.062 0.051 0.035 0.03
p < 0.01 0.079 0.009 0.005 0.007 0.003 0.001 0.004 0.005 0.003 0.003 0
p < 0.001 0.004 0 0.001 0 0 0 0 0 0 0 0
