# Supplementary material for: Discovering biological connections between experimental conditions based on common patterns of differential gene expression
Source: BMC Bioinformatics. 2011 Sep 27;12:381. doi: 10.1186/1471-2105-12-381 (PMC3203354; doi:10.1186/1471-2105-12-381)
Supplement: Additional file 8 — Variation of openSESAME p values with signature composition in GEO series GSE2225 and GSE21653. A, B. For each subset size, 1000 subsets of the up-and down-regulated genes in the signature were obtained by permutation, maintaining a constant signature size, and SA scores were computed using each GEO series. Fisher's exact test or a two-sided Kolmogorov-Smirnov (K-S) test were used to compute p values for each permutation. C, D. The expression values of each gene were shuffled independently 100 times, and for each shuffled dataset, 10 subsets of the up- and down-regulated genes were obtained for each subset size and SA scores and p values were computed. E, F. A total of 100 simulated datasets were obtained by generating random values from a standard normal distribution and z-normalizing each row ("gene") across all columns ("samples"). For each simulated dataset, 10 subsets of the up- and down-regulated genes were obtained for each subset size and SA scores and p values were computed. [file 1471-2105-12-381-S8.PDF]

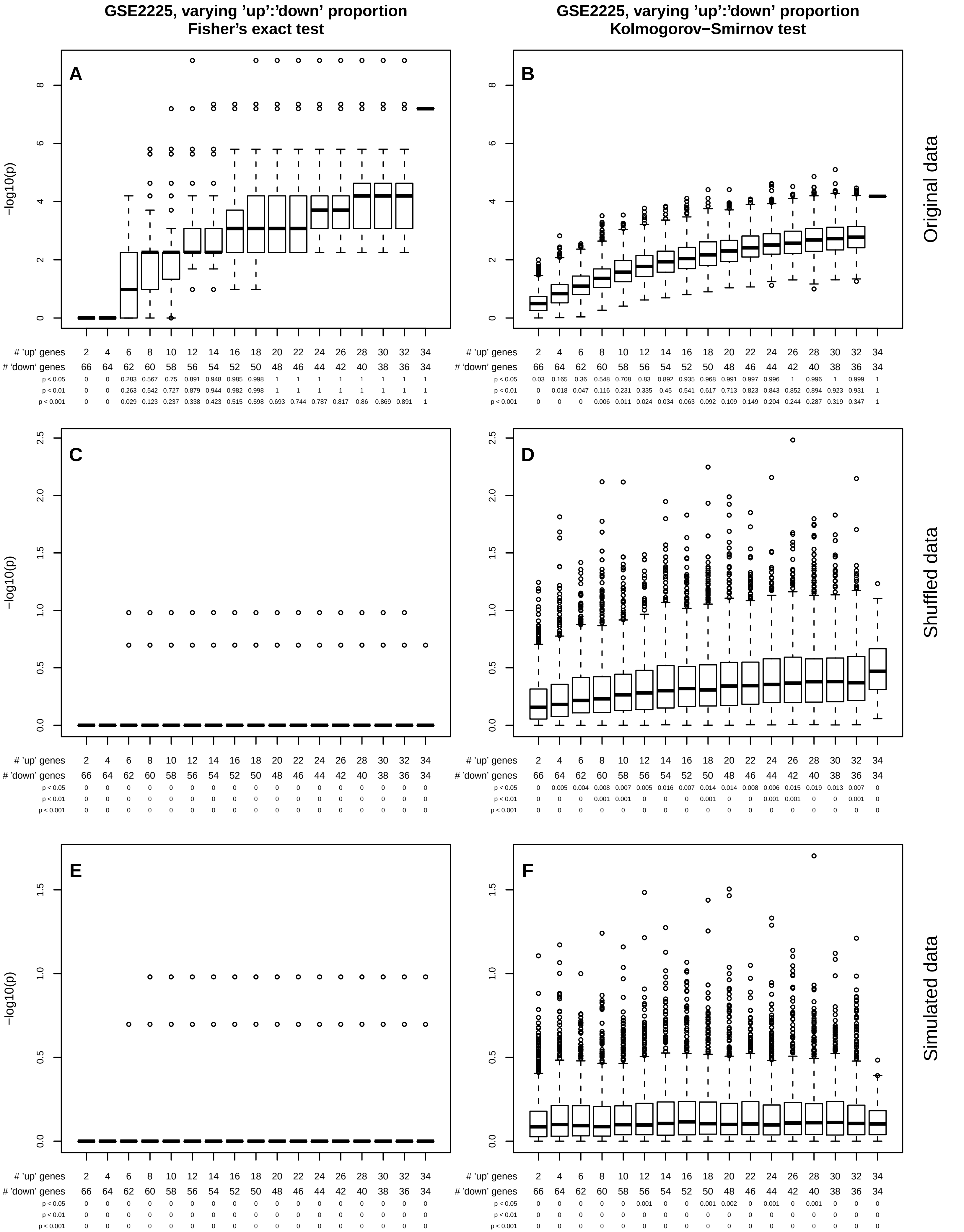
GSE2225, varying 'up':'down' proportion
Fisher's exact test
GSE2225, varying 'up':'down' proportion
Kolmogorov−Smirnov test
Original data
Shuffled data
Simulated data
A
B
C
D
E
F
−log10(p)
# 'up' genes 2 4 6 8 10 12 14 16 18 20 22 24 26 28 30 32 34
# 'down' genes 66 64 62 60 58 56 54 52 50 48 46 44 42 40 38 36 34
p < 0.05 0 0 0.283 0.567 0.75 0.891 0.948 0.985 0.998 1 1 1 1 1 1 1 1
p < 0.01 0 0 0.263 0.542 0.727 0.879 0.944 0.982 0.998 1 1 1 1 1 1 1 1
p < 0.001 0 0 0.029 0.123 0.237 0.338 0.423 0.515 0.598 0.693 0.744 0.787 0.817 0.86 0.869 0.891 1
# 'up' genes 2 4 6 8 10 12 14 16 18 20 22 24 26 28 30 32 34
# 'down' genes 66 64 62 60 58 56 54 52 50 48 46 44 42 40 38 36 34
p < 0.05 0.03 0.165 0.36 0.548 0.708 0.83 0.892 0.935 0.968 0.991 0.997 0.996 1 0.996 1 0.999 1
p < 0.01 0 0.018 0.047 0.116 0.231 0.335 0.45 0.541 0.617 0.713 0.823 0.843 0.852 0.894 0.923 0.931 1
p < 0.001 0 0 0 0.006 0.011 0.024 0.034 0.063 0.092 0.109 0.149 0.204 0.244 0.287 0.319 0.347 1
# 'up' genes 2 4 6 8 10 12 14 16 18 20 22 24 26 28 30 32 34
# 'down' genes 66 64 62 60 58 56 54 52 50 48 46 44 42 40 38 36 34
p < 0.05 0 0 0 0 0 0 0 0 0 0 0 0 0 0 0 0 0
p < 0.01 0 0 0 0 0 0 0 0 0 0 0 0 0 0 0 0 0
p < 0.001 0 0 0 0 0 0 0 0 0 0 0 0 0 0 0 0 0
# 'up' genes 2 4 6 8 10 12 14 16 18 20 22 24 26 28 30 32 34
# 'down' genes 66 64 62 60 58 56 54 52 50 48 46 44 42 40 38 36 34
p < 0.05 0 0.005 0.004 0.008 0.007 0.005 0.016 0.007 0.014 0.014 0.008 0.006 0.015 0.019 0.013 0.007 0
p < 0.01 0 0 0 0.001 0.001 0 0 0 0.001 0 0 0.001 0.001 0 0 0.001 0
p < 0.001 0 0 0 0 0 0 0 0 0 0 0 0 0 0 0 0 0
# 'up' genes 2 4 6 8 10 12 14 16 18 20 22 24 26 28 30 32 34
# 'down' genes 66 64 62 60 58 56 54 52 50 48 46 44 42 40 38 36 34
p < 0.05 0 0 0 0 0 0 0 0 0 0 0 0 0 0 0 0 0
p < 0.01 0 0 0 0 0 0 0 0 0 0 0 0 0 0 0 0 0
p < 0.001 0 0 0 0 0 0 0 0 0 0 0 0 0 0 0 0 0
# 'up' genes 2 4 6 8 10 12 14 16 18 20 22 24 26 28 30 32 34
# 'down' genes 66 64 62 60 58 56 54 52 50 48 46 44 42 40 38 36 34
p < 0.05 0 0 0 0 0 0.001 0 0 0.001 0.002 0 0.001 0 0.001 0 0 0
p < 0.01 0 0 0 0 0 0 0 0 0 0 0 0 0 0 0 0 0
p < 0.001 0 0 0 0 0 0 0 0 0 0 0 0 0 0 0 0 0

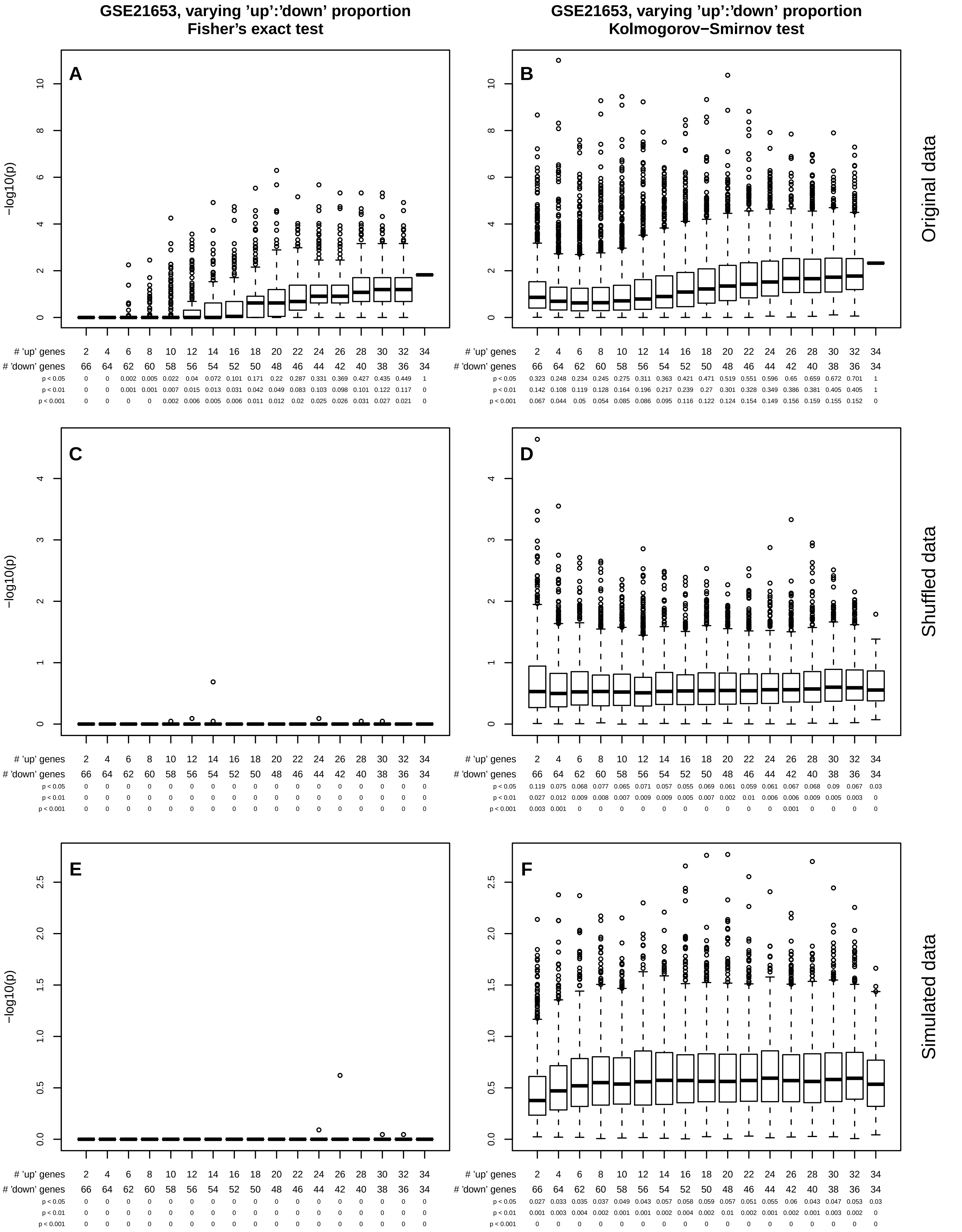
GSE21653, varying 'up':'down' proportion
Fisher's exact test
GSE21653, varying 'up':'down' proportion
Kolmogorov−Smirnov test
A
B
C
D
E
F
−log10(p)
Original data
Shuffled data
Simulated data
# 'up' genes 2 4 6 8 10 12 14 16 18 20 22 24 26 28 30 32 34
# 'down' genes 66 64 62 60 58 56 54 52 50 48 46 44 42 40 38 36 34
p < 0.05 0 0 0.002 0.005 0.022 0.04 0.072 0.101 0.171 0.22 0.287 0.331 0.369 0.427 0.435 0.449 1
p < 0.01 0 0 0.001 0.001 0.007 0.015 0.013 0.031 0.042 0.049 0.083 0.103 0.098 0.101 0.122 0.117 0
p < 0.001 0 0 0 0 0.002 0.006 0.005 0.006 0.011 0.012 0.02 0.025 0.026 0.031 0.027 0.021 0
p < 0.05 0.323 0.248 0.234 0.245 0.275 0.311 0.363 0.421 0.471 0.519 0.551 0.596 0.65 0.659 0.672 0.701 1
p < 0.01 0.142 0.108 0.119 0.128 0.164 0.196 0.217 0.239 0.27 0.301 0.328 0.349 0.386 0.381 0.405 0.405 1
p < 0.001 0.067 0.044 0.05 0.054 0.085 0.086 0.095 0.116 0.122 0.124 0.154 0.149 0.156 0.159 0.155 0.152 0
p < 0.05 0 0 0 0 0 0 0 0 0 0 0 0 0 0 0 0 0
p < 0.01 0 0 0 0 0 0 0 0 0 0 0 0 0 0 0 0 0
p < 0.001 0 0 0 0 0 0 0 0 0 0 0 0 0 0 0 0 0
p < 0.05 0.119 0.075 0.068 0.077 0.065 0.071 0.057 0.055 0.069 0.061 0.059 0.061 0.067 0.068 0.09 0.067 0.03
p < 0.01 0.027 0.012 0.009 0.008 0.007 0.009 0.009 0.005 0.007 0.002 0.01 0.006 0.006 0.009 0.005 0.003 0
p < 0.001 0.003 0.001 0 0 0 0 0 0 0 0 0 0 0.001 0 0 0 0
p < 0.05 0 0 0 0 0 0 0 0 0 0 0 0 0 0 0 0 0
p < 0.01 0 0 0 0 0 0 0 0 0 0 0 0 0 0 0 0 0
p < 0.001 0 0 0 0 0 0 0 0 0 0 0 0 0 0 0 0 0
p < 0.05 0.027 0.033 0.035 0.037 0.049 0.043 0.057 0.058 0.059 0.057 0.051 0.055 0.06 0.043 0.047 0.053 0.03
p < 0.01 0.001 0.003 0.004 0.002 0.001 0.001 0.002 0.004 0.002 0.01 0.002 0.001 0.002 0.001 0.003 0.002 0
p < 0.001 0 0 0 0 0 0 0 0 0 0 0 0 0 0 0 0 0
